# Blue Pyoktanin in the Treatment of Inoperable Malignant Growths*Read before the Georgia Medical Association at Macon, April 21, 1897.

**Published:** 1897-08

**Authors:** Henry R. Slack

**Affiliations:** LaGrange, Ga., Professor of Chemistry and Physics in the Southern Female College, and Lecturer on Physiology


### BLUE PYOKTANIN IN THE TREATMENT OF INOPERABLE MALIGNANT GROWTHS.*

By HENRY R. SLACK, Ph.M., M.D., LaGrange, Ga.,
Professor of Chemistry and Physics in the Southern Female College, and Lecturer on Physiology.

There is no disease to which flesh is heir, that so appeals to the sympathy of the physician, as an inoperable case of cancer. Not even consumption's ghastly form is so dreadful to the patient and hopeless of results to the physician. The sufferer from tuberculosis is almost invariably buoyed up by an ever present, but delusive,

*Read before the Georgia Medical Association at Macon, April 21, 1897.

hope that "as soon as I get rid of this heavy cold I will be all right"; but the poor cancer patient is bereft of hope. He sees the vulture, Prometheus-like, feeding upon his vitals, but has little hope of Hercules coming to the rescue.

Now, what are we to do in such cases? To dismiss them because we cannot promise cures would be inhuman and cowardly.

These patients should be *treated* on the same principles as those unfortunate beings afflicted with chronic nephritis, consumption, or any other disease recognized as incurable.

When the surgeon admits that his knife can promise no hope, we should bring to bear upon the case every other means to make it *yield* to scientific treatment. Even though we may not effect a permanent cure, if we can alleviate the excruciating pains, restore hope, prolong life, and make it more tolerable, we perform a duty worthy of our high calling and receive alike the gratitude of our patient and his friends. To say nothing of that highest of rewards, the approval of our conscience that comes from a knowledge of duty faithfully discharged

Since the work of Professor Von Mosetig, of Vienna, and Dr. Willy Meyer, of New York, with pyoktanin, Dr. Coley by the inoculation of erysipelas, and Adamkiewicz by hypodermic application of neurine, we labor "not as those who have no hope."

In January, 1891, Professor Von Mosetig presented a very instructive paper, entitled "A Contribution to the Treatment of Inoperable Malignant Growths," to the Vienna Society of Physicians, reporting on his experience with blue pyoktanin in such cases. Shortly after (April 25th, 1891) appeared an article from Dr. Willy Meyer in the *Medical Record*, "Notes on the Effect of the Aniline Dyes, Especially Blue Pyoktanin, in the Treatment of Inoperable Malignant Growths." Dr. Meyer also has another article on the same subject in the *Annals of Surgery* for November, 1893. Here I wish to acknowledge my obligations to Dr. Meyer for kindly furnishing me his valuable reprints and a list of the literature of this subject.

Billroth, true to his instincts as a great surgeon, had little faith in the use of pyoktanin, and did not believe it had any specific influence whatever. He claimed that it was merely the water forcibly pressed into the tissues made them swell and unable to live. He reported that three of his patients got worse under its use and one died of sepsis.

Fortunately Von Mosetig and Dr. Willy Meyer had better success, and my experience agrees with theirs, as a report of the following cases will show.

*Case 1.*—October 21, 1896, J. E. C., aged forty-three, white, male, farmer. Father died of cancer. Complains of sore throat and difficulty in swallowing for the last two months. Examination showed a neoplasm size of hickory-nut on the dorsum of the tongue a little left of the median line. Has warty appearance and the papillæ all around are enlarged and indurated. Tumor can be felt from outside, and submaxillary glands indurated.

October 24th. Tumor growing. Pain extends further down throat and deglutition more difficult. Treated with Lugol's solution of iodine and gave gargle of same containing two minims carbolic acid to the ounce.

November 14th. Tumor getting larger, covered with a mucopus and becoming more painful, especially at night. Necessary to use morphine in order to sleep.

November 19th. Complaints that he could not sleep last night as he like to have choked to death. Iodine doing no good, so ordered blue pyoktanin pencil and powder. Dr. F. M. Ridley saw the case in consultation. Decided inoperable and approved of the trial of pyoktanin.

December 8th. Patient has been to Atlanta and consulted six surgeons of that city, including two ex-presidents of this Association, one of whom he heard to remark: "It is cancer. Prognosis bad, not one chance in a hundred from operation, and he won't live till New Year." Necrosis has begun, odor very offensive. The tumor is now as large as a hen's egg and very painful. Can hardly use tongue enough to articulate his words. Treated. Parenchymatous injection of 1.5 c.c. of a two per cent. solution of blue pyoktanin and gave patient pencil to apply daily.

December 12th. Patient says he has not suffered so much as before using paint, and can talk a little plainer. Treated. Injection of 1.5 c.c.

December 19th. Has suffered very little pain. Can talk without difficulty. Necrobiosis has set in and the tumor has diminished considerably. Injected 1.5 c.c.

December 22d. Patient improving. Small nodules beginning to drop off and under them granulating surfaces appear. Reduced about one-fourth. Injected 1.5 c.c.

December 27th. Feels much better. Does not have to use morphine at all. Injected 1.5 c.c.

January 2d, 1897. Improvement continues. Tumor not one-half as large as when I began parenchymatous injections. Injected 1.5 c.c.

January 9th. Patient feels much relieved. Cachexia nearly all gone. Fetid odor no longer present. Appetite returning and anxious to try solid food, which was allowed. Injected.

January 12th. Tumor has reduced to less than one-fourth of the original size. Patient eats solids and swallows without difficulty. Color good, and strength rapidly returning. Injected.

January 16th. Improvement continues. Injected.

January 23d. Feels no inconvenience from neoplasm, which is now only one-sixth of the original size. Says, "I am about well now." From then until February 28th he only received one injection weekly. I was called to New Orleans and was absent for two weeks, during which time patient took cold and I found on my return the neoplasm had grown some. Gave two injections weekly and necrobiosis at once began.

April 17th. Tumor diminishing in size, though somewhat painful. Pain was relieved by injection of the pyoktanin solution.*

*Case 2.*—January 23d, 1897, R. H., aged thirty-seven, colored, female. Has had seven children, no miscarriages. Family history negative. Now has a four months' baby, and since birth of this child lochia has continued. During first four months of pregnancy had occasional hemorrhages. Patient fairly well nourished, though much weaker than after birth of other children. Prescribed injection of bichloride mercury 1 to 4,000 and aromatic

*June 18. Case still under treatment and doing very well. Attends to all his duties on farm, and except for difficulty in swallowing cold water suffers little inconvenience.

sulphuric acid and elixir calisaya as an astringent tonic and told her to return in a week prepared for an examination.

February 3d. Patient said, "Do not bleed so much, but still have bad smelling discharge." Examination showed a large ulcerated cauliflower neoplasm enveloping the entire cervix and extending well up the posterior portion of the body of the uterus. It was as large as a small orange and bled freely when touched. Dr. Cason saw case with me and agreed that it was an inoperable carcinoma.

Treatment: Washed out vagina and off the neoplasm with solution bichloride mercury 1 to 2,000 and gave parenchymatous injection of 2 c.c. of a two per cent. solution of pyoktanin. I also gave the patient wash of bichloride mercury to be used daily, after which she uses 6 c.c. of one per cent. solution pyoktanin.

February 9th. All hemorrhage has ceased and patient feels better. Injected 2 c.c.

February 12th. Discharge not so offensive as before; pain has diminished. Injected 2 c.c.

February 16th. Improvement. Necrobiosis established, and nodules are supported by smaller peduncles. Injected.

February 19th. Improvement continues. Injected.

February 23d. Patient says when she injects the bichloride mercury wash small pieces of flesh size of rice to pea come out. While treating a nodule size of large pea dropped off. Surface presents healthier appearance.

I have continued parenchymatous injections of 2 c.c. of the pyoktanin twice a week ever since. During my absence Dr. Cason kindly continued treatment and has since seen it several times. The size of the tumor is steadily diminishing and the patient now (April 17th) suffers absolutely no inconvenience.

*Case 5.* April 6th, 1897. Mrs. S. A. J., aged forty-seven; white. Had eight children, youngest three years. Family history negative. First trouble occurred when nursing last child. Three years ago noticed lump in right breast size of a marble that grew steadily. Breast was removed in May, 1895, and all healed up but a little place size of pea, but this remained hard and in October began to ulcerate. Poorly nourished woman with recurrent cancer of right breast. Open ulcerating wound 10 cm. long by 3.5 cm. wide, eroded edges. Erosions being in some places 2 cm. deep. The whole wound is covered with necrotic tissue and the cavities filled with a fetid cancerous secretion. The glands of the axillary region are indurated, painful, and cancerous nodules depend from the wound near the axilla. The induration extends up to the clavicle and patient complains of pain in neck and on moving arm.

Treated: Cleansed wound out thoroughly with hydrogen peroxide and then bichloride mercury. Gave parenchymatous injection of 2 c.c. of the 2 per cent. solution pyoktanin and then dusted the wound over thoroughly with a two per cent. powder.

April 10th. Has not suffered so much since using paint, and sleeps better. Treated.

April 13th. Rests easier now and has better use of arm. Treated.

April 17th. Complains of some pain, but after the injection this disappeared. There is softening in the injected area and general appearance of wound and patient has improved.

I have two other patients under treatment; one, cancer of the womb, and the other of the breast, and both are improving. There seems to be a reasonable probability of the ulcerating uterus healing; at least the improvement since I began treatment with pyoktanin (March 15th) is marked.*

Now, a word as to the technique of the treatment which varies with the location.

The injection should be made under strict aseptic and antiseptic precautions. The skin where the needle is to enter should be thoroughly cleansed with bichloride solution.

The needles may be long, short or curved; but must not be too small caliber, and should be boiled after using.

I used the large hypodermic syringe and injected from 1 to 2 c.c.

*Three of these cases are still (June 15th) under treatment, and are progressing very nicely. They suffer almost no pain, general health is good, the malignant growths are held well in check, and one is apparently getting well. Two have not been treated for forty days. They suffer the characteristic excruciating cancer pains, general health has begun to fail, and the growths have made marked progress.

of a two per cent. solution. This is more than twice as strong as recommended by Dr. Meyer and Von Mosetig.

The patient is given the pyoktanin pencil, one per cent. solution or a two per cent. power, as the case may require, to apply daily.

Thus far have seen no untoward effects from its use. I always employ Merck's because I can rely on its purity.

The only objection to it is staining everything with which it comes in contact, but what is the soiling of linen when compared with the following advantages?

1st. Its analgesic effects are marked, as patients soon rest easily without the aid of morphine.

2d. "The improvement of the function of the part involved." The man who could hardly speak so as to be understood talked without difficulty after the third injection.

3d. The improvement in general health which has taken place in all five of my cases.

4th. The element of hope that is added to the life of suffering man, brightening the remainder of life.

While I do not claim to have cured my patients, still I have relieved their pains, and rendered them less burdensome to themselves and their friends.

I agree with Dr. Meyer in Von Mosetig's conclusions, "that it has been proved by practice, that parenchymatous injections of inoperable malignant growths with pyoktanin can produce disappearance of malignant tissue—though in exceptional cases, and can heal neoplastic ulcerations."

Pyoktanin when properly used is certainly a palliative treatment for cancer that deserves an honest hopeful trial, for by its use many have been relieved and some cured.

---

A society has been organized in the University of Chicago, the members of which will bequeath their brains to the society, as well as write out a truthful, conscientious, and, so far as possible, unbiased history of their mental processes from the beginning to the end of their life.—*Lancet-Clinic.*

